# Supplementary material for: SERINC5 Restricts HIV-1 Infectivity by Promoting Conformational Changes and Accelerating Functional Inactivation of Env
Source: Viruses. 2022 Jun 25;14(7):1388. doi: 10.3390/v14071388 (PMC9323560; doi:10.3390/v14071388)
Supplement: Supplementary file 1 [file viruses-14-01388-s001.zip › viruses-1742859-supplementary.pdf]

Fig S1

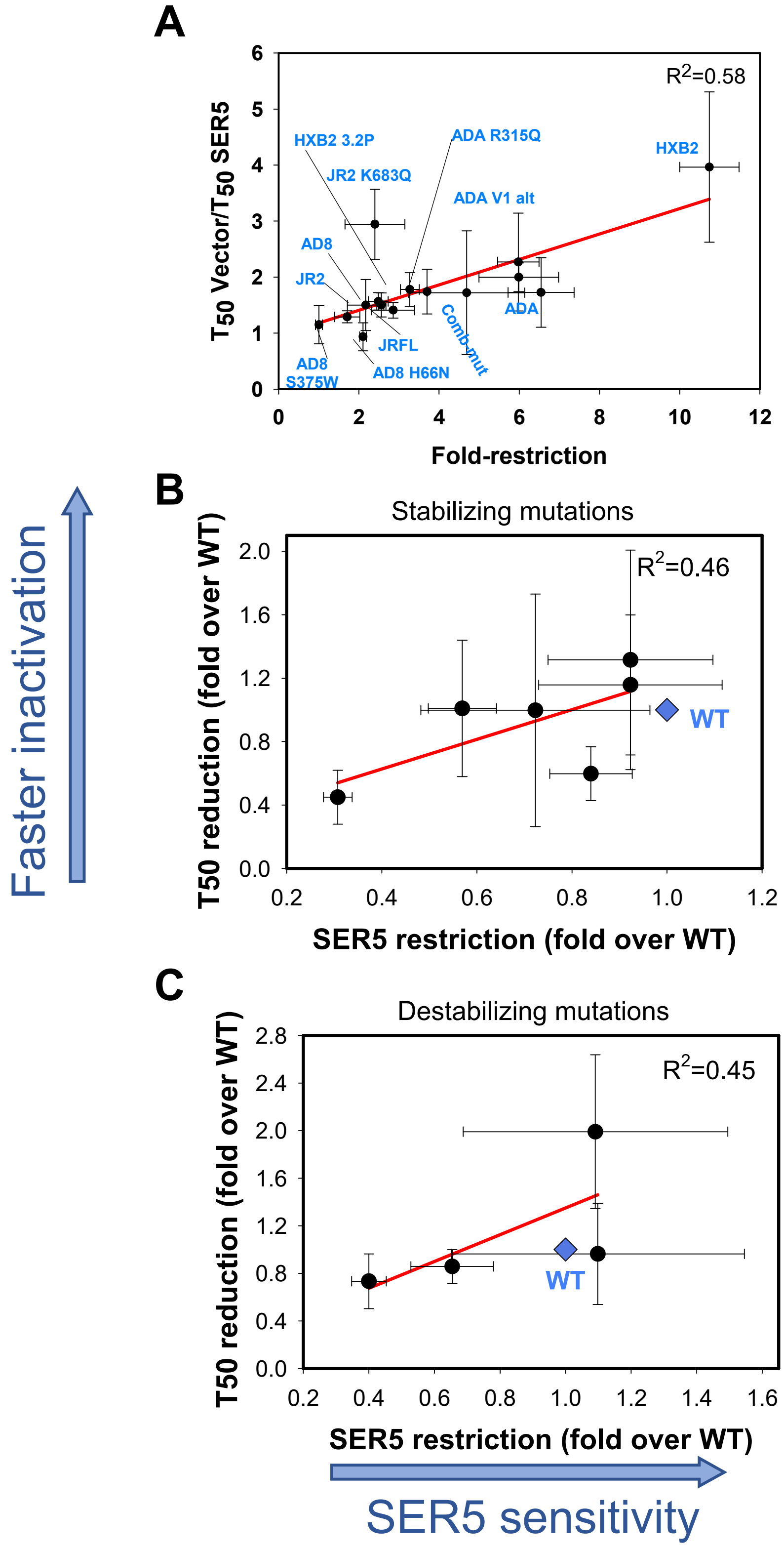

$$\text{T50 reduction} = \frac{T50_{Vec}{}^{mut}\ /T50_{SER5}{}^{mut}}{T50_{Vec}{}^{WT}/T50_{SER5}{}^{WT}}$$

$$\text{SER5 restriction} = \frac{FR_{Vec}{}^{mut}\ /FR_{SER5}{}^{mut}}{FR_{Vec}{}^{WT}/FR_{SER5}{}^{WT}}$$

Figure S1. Correlation between the rates of spontaneous inactivation and fold-restriction by SER5 for wild-type and mutant HIV-1 Envs. (A) Scatter plot showing SER5-mediated acceleration of Env inactivation (T_{50}Vector/T_{50}SER5) *vs* SER5 fold-restriction for 5 wild-type (WT) Envs and 10 Env mutants. A linear regression is shown by red line. The data are means and S.D. of 2 to 4 independent experiments, each performed in triplicate. The values of T_{50} ratio, fold-restriction, and the numbers of independent experiments are listed in Table S1. (B) and (C) Scatter plots of the fold-T_{50} reduction by SER5 *vs* SER5 restriction for HIV-1 Envs containing stabilizing mutations (B) or destabilizing mutations (C). For the combined scatter plot for all mutations, see Fig. 2B. The WT values across different Env strains were set to 1 (blue symbol). The red line is linear regression. The blue arrows along the axes show shifts from the regression line corresponding to SER5 sensitization. Data are means and S.D. from 2 to 4 independent experiments, each performed in triplicate. The number of experiments, T_{50} ratio, and fold-restriction values are listed in Table S1. The formulas used to calculate the values plotted in A and B are given below the graphs. Abbreviations: *Vec,* vector, *mut,* mutant, WT, wild-type, and FR, fold-restriction.

Fig S2

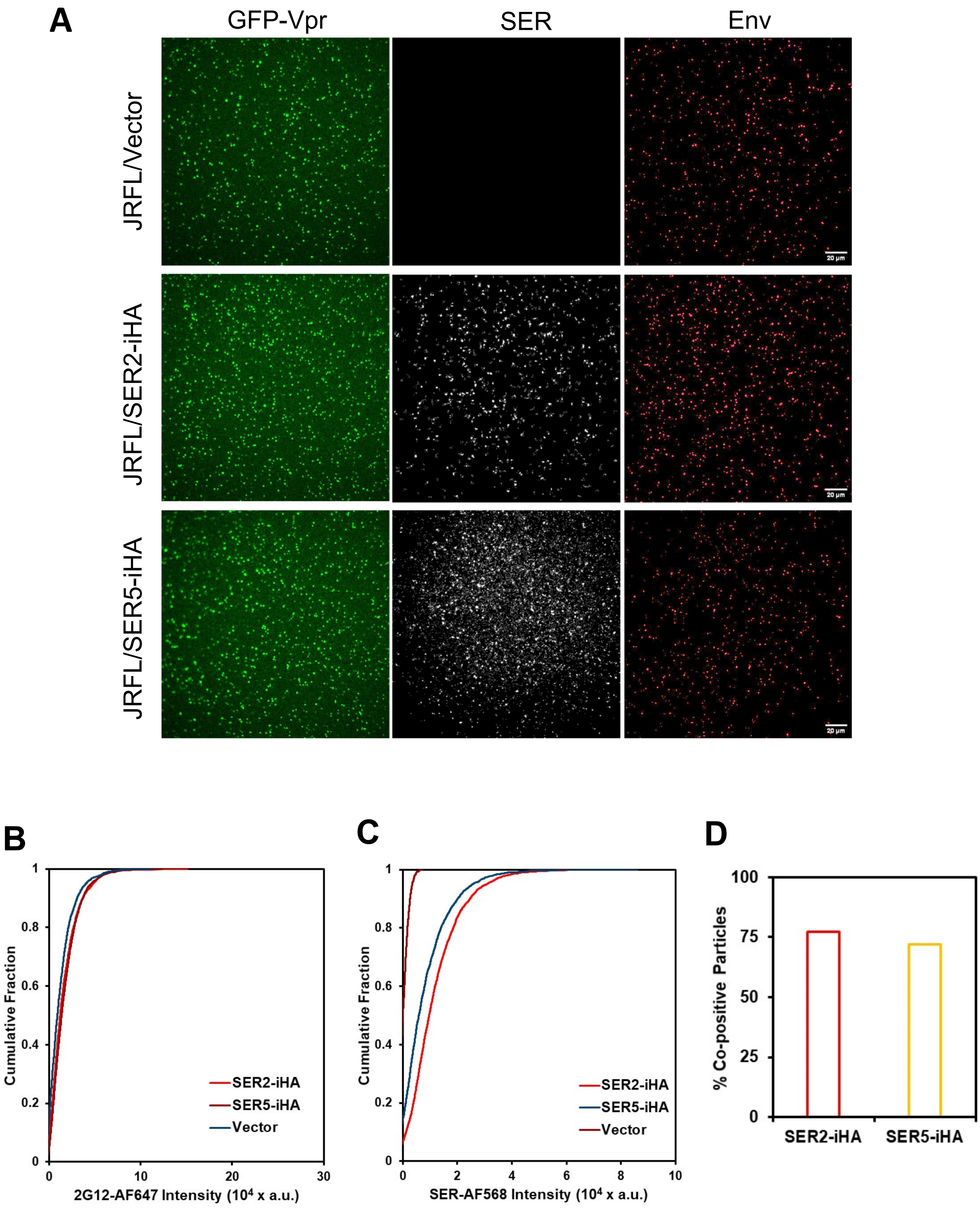
A
GFP-Vpr
SER
Env
JRFL/Vector
JRFL/SER2-iHA
JRFL/SER5-iHA
20 µm
B
Cumulative Fraction
SER2-iHA
SER5-iHA
Vector
2G12-AF647 Intensity (10^4 x a.u.)
C
Cumulative Fraction
SER2-iHA
SER5-iHA
Vector
SER-AF568 Intensity (10^4 x a.u.)
D
% Co-positive Particles
SER2-iHA
SER5-iHA

Figure S2. Characterization of pseudoviruses lacking or containing SER-iHA. HIV-1 JRFL pseudoviruses labeled with GFP-Vpr and lacking or containing SER2-iHA or SER5-iHA were adhered to poly-D-lysine coated glass chambered coverslips, fixed, and immunostained with anti-gp120 2G12 human (Env) (A, B) and anti-HA (SERINC) mouse (A, C) antibodies, followed by staining with anti-human-AF647 and anti-mouse-CF568 antibodies, respectively. (A) Images of GFP-Vpr containing pseudoviruses stained for Env and SERINC-iHA. (B) Plot of cumulative fraction of fluorescence intensities of particles positive for Env. (C) Plot of cumulative fraction of fluorescence intensities of particles positive for SER-iHA. (D) Co-localization of JRFL Env and SER on viral particles. Around 2,250 single viruses were imaged and analyzed using a custom-written Matlab protocol (B-D).

Fig. S3

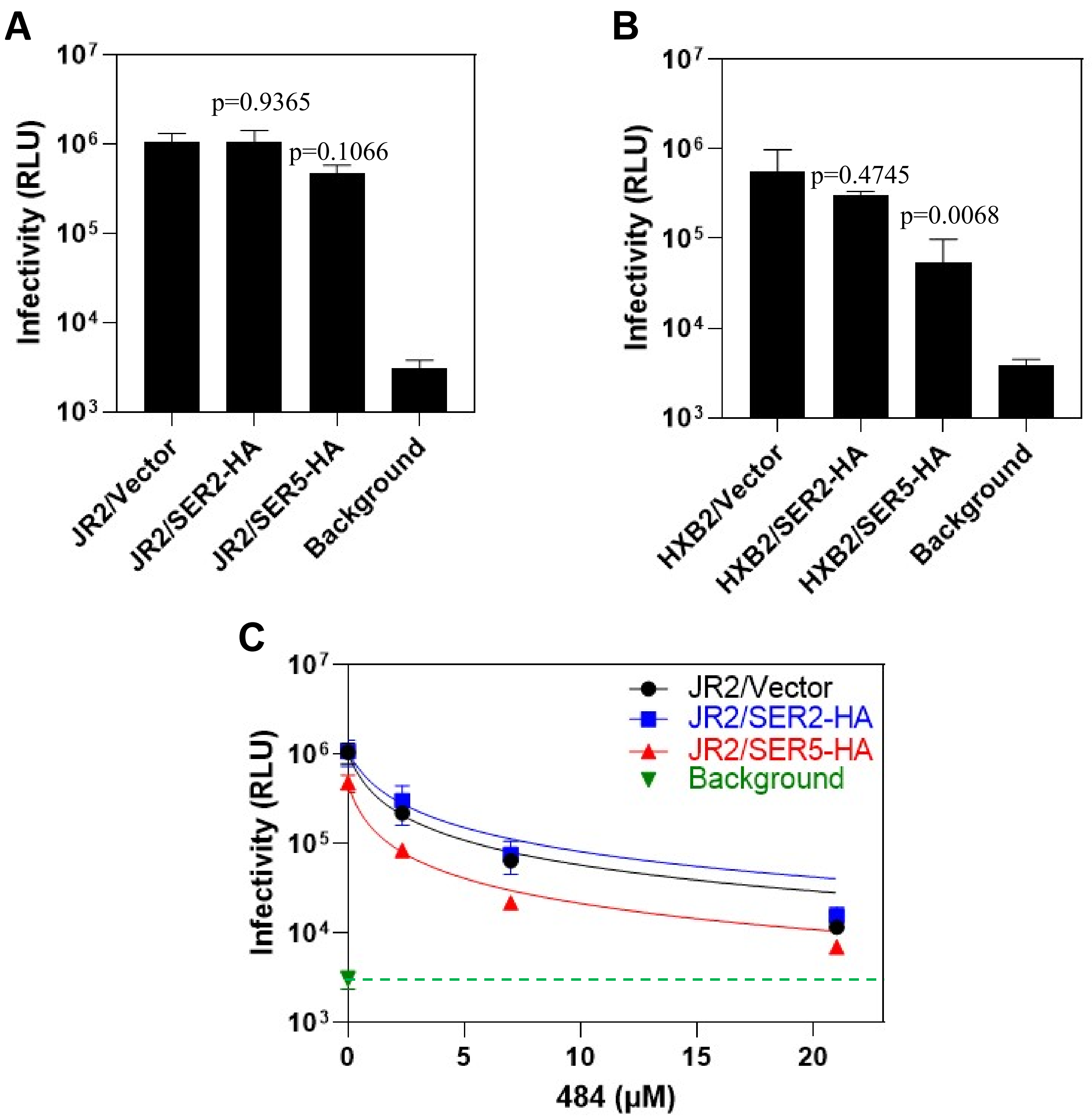

Figure S3. Raw infectivity data using a luciferase assay for pseudoviruses lacking or containing SER-HA. This figure is related to main Figure 4. Infectivity in TZM-bl cells plotted as relative light units (RLU) for JR2 (A) and HXB2 (B) pseudoviruses in the absence of **484** compound. Data are means and S.D. of 2 (JR2 pseudoviruses), 3 (HXB2/SER5-HA), and 2 (HXB2/SER2-HA) independent combined experiments, each performed in triplicate. Statistical significance (Student's unpaired t test) is shown above the columns. (C) Raw infectivity data for JR2 pseudoviruses pre-treated with **484.** Data are means and S.D. of 2 independent combined experiments, each performed in triplicate. Green inverted triangle and dashed line indicate the background level. The IC_{50} values obtained by curve-fitting and statistical significance are the same as in Fig. 4A.

n	Env	T_{50} (h)	SD	p-value (t-test)	T_{50} Ratio (Vector/ SER5)	T_{50} (h)	SD	p-value (ANOVA RM)	T_{50} Ratio (Vector/SER)	Fold restriction	Phenotype	Representative experiment	Combined experiments
4	HXB2/Vector	4.7	0.81		4 ± 1.3	4.7	0.01		4.4 ± 0.2	10.7 ± 0.74	Tier-1 like	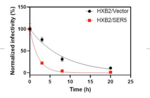	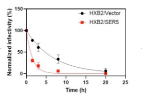
	HXB2/SER5	1.2	0.35	0.0002		1.1	0.05	<0.0001					
2	HXB2/Vector	3.3	0.07		1.3 ± 0.1	3.3	0.02		1.3 ± 0.1	0.6 ± 0.01	Tier-1 like	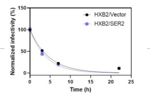	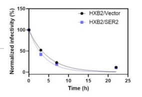
	HXB2/SER2	2.6	0.27	0.0711		2.6	0.03	0.0851					
2	HXB2 3.2P/Vector	12.7	1.65		1.8 ± 0.3	12.7	0.01		1.8 ± 0.01	3.3 ± 0.23	Resistant to Abs and cold	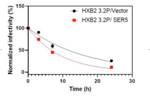	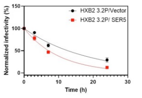
	HXB2 3.2P/SER5	7.1	0.77	0.0494		7.1	0.05	0.0373					
3	ADA/Vector	5.1	1.31		1.7 ± 0.6	5	0.01		1.7 ± 0.01	6.5 ± 0.82	Tier-1 like	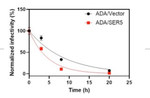	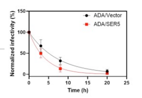
	ADA/SER5	2.9	0.74	0.069		2.9	0.02	0.0795					
2	ADA-V1 alt/Vector	11.1	2.98		2.3 ± 0.9	10.4	0.01		2.3 ± 0.01	6.0 ± 0.52	N139/I140 deletion, N142S	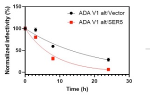	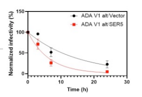
	ADA-V1 alt/SER5	4.9	1.34	0.116		4.4	0.02	0.0865			GP120 V1 stabilizing		
2	ADA-N302Y/Vector	7.3	0.27		2.0 ± 0.3	7.3	0.006		2.0 ± 0.01	6.0 ± 0.99	GP120 V3 Stabilizing	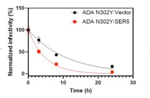	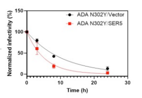
	ADA-N302Y/SER5	3.7	0.45	0.0103		3.7	0.02	0.0112					
2	ADA-R315Q/Vector	8.4	0.13		1.8 ± 0.4	8.4	0.01		1.7 ± 0.005	3.7 ± 0.01	GP120 V3 Stabilizing	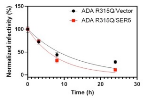	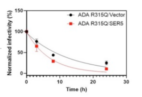
	ADA-R315Q/SER5	4.8	1.11	0.0451		4.8	0.01	0.0719					
2	Comb-mut/Vector	14.3	5.2		1.7 ± 1.1	13.7	0.005		1.7 ± 0.003	4.7 ± 1.45	8 Stabilizing mutations on ADA	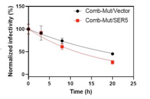	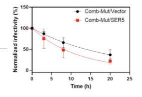
	Comb-mut/SER5	8.3	4.4	0.3386		8.1	0.01	0.3786			More thermostable		
3	JRFL/Vector	12.2	1.75		1.5 ± 0.2	12.1	0.004		1.5 ± 0.0	2.6 ± 0.11	Tier-2 like	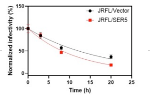	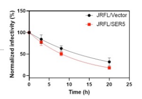
	JRFL/SER5	8.1	0.1	0.0153		8.1	0.004	0.0205					
2	JRFL-I423A/Vector	8.3	0.3		1.3 ± 0.1	8.3	0.01		1.3 ± 0.0	1.7 ± 0.32	Induced open structure (β20-β21 sheet)	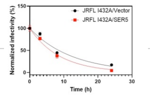	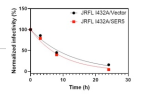
	JRFL-I423A/SER5	6.4	0.5	0.0427		6.4	0.01	0.0435			Sensitive to CD4mc, sCD4, Abs		
3	JR2/Vector	11.8	1.14		1.5 ± 0.5	11.8	0.006		1.5 ± 0.002	2.2 ± 0.45	Tier-2 like	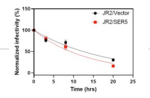	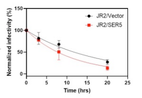
	JR2/SER5	7.9	2.3	0.0547		7.7	0.01	0.0818					
3	JR2-F673L/Vector	12.4	2.40		1.4 ± 0.5	12.3	0.01		1.4 ± 0.002	2.4 ± 0.8	MPER destabilizing	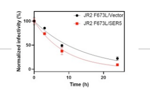	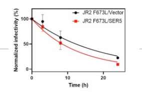
	JR2-F673L/SER5	8.5	2.17	0.1106		8.5	0.01	0.1334					
3	JR2-K683Q/Vector	17.7	1.4		2.9 ± 0.6	17.7	0.004		2.9± 0.006	2.4 ± 0.74	MPER destabilizing	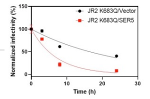	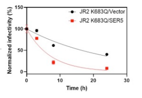
	JR2-K683Q/SER5	6	1.2	0.0004		6	0.01	0.001					
2	AD8/Vector	13.7	1.14		1.6 ± 0.1	13.7	0.003		1.6 ± 0.01	2.5 ± 0.24	Tier-2 like	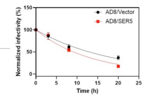	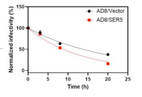
	AD8/SER5	8.8	0.5	0.0296		8.8	0.005	0.0444					
2	AD8-S375W/Vector	14	1.05		1.1 ± 0.3	14	0.007		1.2 ± 0.001	1.0 ± 0.09	More CD4-bound-like	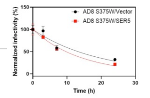	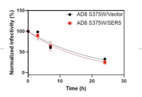
	AD8-S375W/SER5	12.2	3.5	0.5548		12.1	0.006	0.5694			Exposure of CoR binding site, Cold sensitive		
2	AD8 H66N/Vector	13.5	1.68		0.9 ± 0.2	12.5	0.006		0.8 ± 0.0	2.1 ± 0.08	More closed conformation	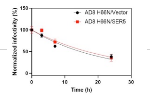	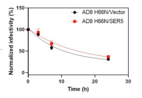
	AD8 H66N/SER5	14.4	3.39	0.765		15.8	0.004	0.1662			Cold resistant		
2	VSV/Vector	12.2	4.7		1.2 ± 0.5	11.9	0.01		1.1 ± 0.001	2.2 ± 0.82	non HIV G protein expression	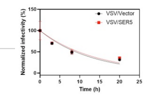	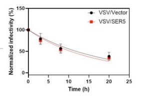
	VSV/SER5	10.5	1.2	0.6568		10.5	0.01	0.7187					

Table S1. Effect of virion-incorporated SER5 on the rate of infectivity decay of HIV-1 pseudoviruses (T_{50}) and on end-point reduction of infectivity (fold-restriction). SER5-susceptible Env strains are shown in red, while resistant strains, are in black. Fold-restriction is the ratio of infection between control and SER5 containing viruses. The number of independent experiments (n), each performed in triplicate, is listed in the first column. The T_{50} results were analyzed in two ways. (i) The individual T_{50} from independent experiments were averaged and statistical analysis was done using the Student's unpaired t test. (ii) The mean of data points from independent experiments and S.D. were plotted and T_{50} values determined by single-exponential curve fit. Two-way ANOVA repeated measures statistical analysis was done on the raw data (light orange highlighted columns). The phenotypes of Env mutants, as well as representative and combined original data are shown on the right. The significant p-values are listed in blue.

n	Env	484 IC_{50} (µM)	SD	p-value t-test	IC_{50} Ratio (Vector/SER)	484 IC_{50} (µM)	SD	p-value SER *vs* Vector (ANOVA)	p-value SER5 *vs* SER2 (ANOVA)	IC_{50} Ratio (Vector/SER)	Fold restriction
3	HXB2/Vector	31.0	6.83			30.5	2.38				
2	HXB2/SER2	30.1	7.46	0.8985	1 ± 0.3	29.7	2.72	0.8578		1 ± 0.1	1 ± 0.01
3	HXB2/SER5	14.9	4.96	0.0299	2.1 ± 0.8	14.6	2.30	0.016	0.0422	2.1 ± 0.4	11.6 ± 1.5
3	ADA/Vector	11.4	2.72		1.6 ± 0.3	11.1	0.93			1.7 ± 0.2	5.5 ± 2.3
	ADA/SER5	6.9	1.55	0.0694		6.7	0.73	0.0765			
2	HXB2 3.2P/Vector	17.5	7.85		1.9 ± 1.3	16.2	0.93			1.9 ± 0.2	3.3 ± 2.3
	HXB2 3.2P/SER5	9.0	4.23	0.3087		8.3	0.73	0.29			
2	JR2/Vector	0.6	0.09			0.6	0.04				
	JR2/SER2	0.8	0.15	0.2391	0.7 ± 0.2	0.8	0.07	0.2366	0.0939	0.7 ± 0.1	1 ± 0.07
	JR2/SER5	0.5	0.06	0.2359	1.3 ± 0.3	0.5	0.03	0.2429		1.2 ± 0.1	2.2 ± 0.08
2	AD8/Vector	8.9	3.69		1.9 ± 0.8	8.6	1.06			1.8 ± 0.2	2.2 ± 0.6
	AD8/SER5	4.6	0.01	0.2438		4.6	0.20	0.2024			
2	JRFL/Vector	0.6	0.08		1.3 ± 0.2	0.6	0.03			1.3 ± 0.1	2.6 ± 0.6
	JRFL/SER5	0.5	0.05	0.1828		0.5	0.03	0.1685			

Table S2. Effect of virion-incorporated SER5 on sensitivity of HIV-1 Env glycoproteins to 484, a small molecule inhibitor of HIV-1 infection. SER5-sensitive Env strains are shown in red, whereas resistant strains are shown in black. The IC_{50} was calculated using single site binding curve fit. Analysis of the data was done as explained in the legend to Table S1. The significant p-values are listed in blue.
